# 3D Plotting using Camphene as Pore-regulating Agent to Produce Hierarchical Macro/micro-porous Poly(ε-caprolactone)/calcium phosphate Composite Scaffolds

**DOI:** 10.3390/ma12172650

**Published:** 2019-08-21

**Authors:** Jae-Won Choi, Woo-Youl Maeng, Young-Hag Koh, Hyun Lee, Hyoun-Ee Kim

**Affiliations:** 1 School of Biomedical Engineering, Korea University, Seoul 02841, Korea; 2 Department of Materials Science and Engineering, Seoul National University, Seoul 08826, Korea

**Keywords:** Porous scaffolds, 3D printing, poly(ε-caprolactone), hydroxyapatite, in vitro bioactivity

## Abstract

This study demonstrates the utility of camphene as the pore-regulating agent for phase separation-based 3D plotting to produce hierarchical macro/micro-porous poly(ε-caprolactone) (PCL)–calcium phosphate (CaP) composite scaffolds, specifically featuring highly microporous surfaces. Unlike conventional particulate porogens, camphene is highly soluble in acetone, the solvent for PCL polymer, but insoluble in coagulation medium (water). In this study, this unique characteristic supported the creation of numerous micropores both within and at the surfaces of PCL and PCL–CaP composite filaments when using high camphene contents (40 and 50 wt%). In addition, the incorporation of the CaP particles into PCL solutions did not deteriorate the formation of microporous structures, and thus hierarchical macro/micro-porous PCL–CaP composite scaffolds could be successfully produced. As the CaP content increased, the in vitro biocompatibility, apatite-forming ability, and mechanical properties (tensile strength, tensile modulus, and compressive modulus) of the PCL–CaP composite scaffolds were substantially improved.

## 1. Introduction

Poly(ε-caprolactone) (PCL) is one of the most widespread biocompatible and biodegradable polymers, since it can have excellent mechanical properties, including high ductility due to its semi-crystalline structure and low glass transition temperature (~T_g_ = ~ −60 °C) [1,2,3]. In addition, when formulated into porous structures, porous PCL can be used as the scaffold for the repair and regeneration of diseased and/or damaged bone tissues, and is able to induce bone ingrowth into its macropores [4,5]. 

Additive manufacturing (AM) and 3D printing techniques have demonstrated particular promise as manufacturing processes because they can tightly control the external shape and internal porous structure of the porous polymer scaffolds [6,7,8]. Consequently, porous polymer scaffolds produced using these techniques can provide excellent biological functions not only in vitro but also in vivo, with significantly enhanced mechanical properties [9,10,11,12]. Extrusion-based AM techniques, such as fused deposition modelling (FDM) [13,14,15,16] and the 3D plotting of polymer melts [17,18,19,20,21] and solutions [22,23,24], have been widely applied to the production of porous PCL scaffolds due to their ease of manufacture with inexpensive 3D printers. These techniques sequentially deposit PCL filaments extruded through a fine nozzle according to predetermined building paths in a layer-by-layer fashion, allowing the construction of three-dimensionally interconnected pore networks according to a controlled pattern. In addition, several approaches to creating micropores in polymer filaments have been proposed, and can be used to mimic the hierarchical macro/micro-porous architectures of natural bones [25,26,27]. For example, through the FDM process the use of porogen-containing feedstocks can create micropores, since the porogens in the extruded filaments can be subsequently removed by solvent leaching [28,29,30]. However, this process often results in relatively low porosities and limited pore interconnectivity. On the other hand, cryogenic 3D plotting can freeze extruded polymer filaments at cryogenic temperatures and thus create micropores through the removal of the frozen solvent crystals by freeze drying [31,32,33,34,35,36,37,38]. This technique is very useful for producing hydrogel scaffolds with high porosities. However, PCL scaffolds are rarely produced because very low temperatures are required to freeze PCL solutions. Phase separation-assisted 3D plotting techniques can make microporous polymer filaments by extruding polymer solutions in a coagulation bath at room temperature, in which micropores are created thorough the phase separation of the polymer solutions induced by the exchange of the solvent and nonsolvent [39,40,41,42]. This technique has been successfully utilized to produce macroporous PCL scaffolds comprised of microporous filaments. However, relatively dense layers are generally formed on the surfaces of macroporous PCL filaments, thus hindering the mass transport necessary for fast bone regeneration. 

**Figure 1:**
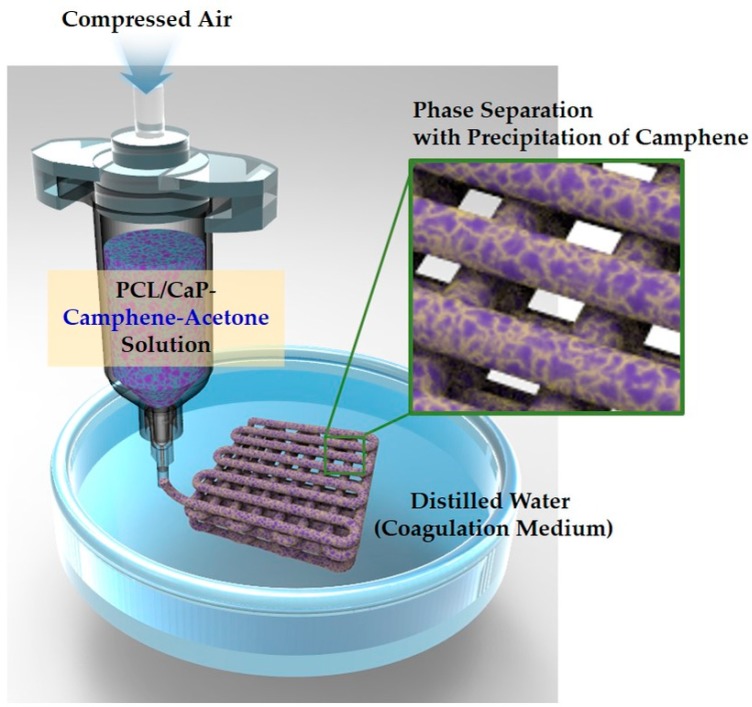
Schematic diagram of the proposed 3D plotting technique using camphene as the pore-regulating agent for the production of hierarchical macro/micro-porous poly(ε-caprolactone) (PCL)/calcium phosphate (CaP) composite scaffolds.

Herein, we propose solid camphene as a novel pore-regulating agent for the phase separation-assisted 3D plotting technique, which can modify the phase separation behavior of PCL phase from a PCL solution, thus allowing for the creation of microporous PCL filaments specifically with microporous surfaces instead of dense skins. Camphene has been widely utilized as a freezing vehicle and porogen for the manufacturing of porous ceramics, where molten camphene at high temperatures (>60 °C) in ceramic suspensions can be crystallized after freeze-casting at room temperature, thus leaving pores after sublimation [43,44,45,46,47]. However, our approach can make full use of the recrystallization of camphene dissolved in acetone used as the solvent for PCL when immersed in a coagulation medium (water). More specifically, when 3D plotted in water, PCL phase can be separated from a PCL–camphene–acetone solution through the exchange of the solvent (acetone) and nonsolvent (water), while, at the same time, the liquid camphene can be recrystallized due to the extraction of acetone from the solution (Figure 1). This unique phase separation behavior coupled with the precipitation of camphene can allow for the creation of micropores throughout the filaments without dense skin layers.

Calcium phosphate (CaP) ceramic is used as the reinforcement for enhanced bone regeneration capability and improved mechanical properties [3,5]. In this study, the effect of camphene content on the development of the micropores in PCL and PCL–CaP composite filaments was characterized. In order to evaluate the potential of the hierarchical macro/micro-porous PCL–CaP composite scaffolds for bone scaffold applications, their microporous structures, biological properties (i.e., in vitro biocompatibility and apatite-forming ability), and mechanical properties were examined.

## 2. Materials and Methods 

### 2.1. PCL and PCL–CaP Solutions Preparation

Unless otherwise specified, all reagents were purchased from Sigma-Aldrich (St. Louis, MO, USA). PCL solutions at a concentration of 22 wt% were prepared by completely dissolving PCL pellets (M_n_ = 80,000) in acetone at 60 °C by magnetic stirring for 3 h. After this, predetermined amounts of solid camphene (0, 20, 30, 40, and 50 wt% in relation to the PCL) were added to the PCL solutions, and then mixed at 60 °C by magnetic stirring for 1 h to prepare clear PCL–camphene–acetone solutions. In addition, calcium phosphate (CaP) powder (Gyeonggi-do, Korea) was used to form PCL–CaP composite solutions. The as-received CaP was composed of hydroxyapatite (HA, Ca_10_(PO_4_)_6_(OH)_2_) and β-tricalcium phosphate (β-TCP, β-Ca_3_(PO_4_)_2_) with a weight ratio of 60:40 (manufacturer’s data report). Predetermined amounts of the CaP powder (10 and 20 wt% in relation to the PCL) were added to the PCL–camphene–acetone solutions with a camphene content of 50 wt%, followed by magnetic stirring for 1 h. Prior to the 3D plotting process, the prepared PCL and PCL–CaP solutions were cooled to room temperature.

### 2.2. 3D Plotting Process

Hierarchical macro/micro-porous PCL and PCL–CaP composite scaffolds were produced by the 3D plotting process (see Figure 1). The PCL solutions were extruded through a nozzle with a diameter of ~500 μm in the distilled water used as the coagulation medium at room temperature, then deposited at a constant speed of 3 mm/s using a computer-controlled robot (Ez-ROBO5, Iwashita, Japan). To achieve uniform PCL filaments, different air pressures were applied to different PCL–camphene solutions, as summarized in Table 1. 

The extruded filaments were deposited at a constant filament distance of 1000 μm and a stacking sequence of 0°/90°. The green scaffolds were freeze-dried for 6 h to remove the frozen camphene, and thus hierarchical macro/micro-porous PCL scaffolds were produced. In addition, two types of PCL–CaP composite scaffolds were produced using PCL–CaP solutions with different CaP contents (10 and 20 wt%). 

### 2.3. Hierarchical Porous Structure, Crystalline Phase, and Chemical Structure Evaluations

The macro/micro-porous structure of the PCL and PCL–CaP composite scaffolds were characterized by optical microscopy and field emission scanning electron microscopy (FE-SEM; JSM-6701F; JEOL Techniques, Tokyo, Japan). The internal and surface structures of the PCL and PCL–CaP composite filaments were examined by FE-SEM. The crystalline phases of PCL–CaP composites were examined by X-ray diffraction (XRD; M18XHF-SRA, MacScience Co., Yokohama, Japan). The chemical structures of the PCL scaffold and camphene were characterized by Fourier-transform infrared spectroscopy (FT-IR; Spectrum 100, Perkin Elmer, USA)

### 2.4. In Vitro Biocompatibility Evaluation

The in vitro biocompatibilities of the hierarchical macro/micro-porous PCL and PCL–CaP composite scaffolds were evaluated using a pre-osteoblast cell line (MC3T3-E1; ATCC, CRL-2593, Rockville, MD, US) [48]. For these tests, porous scaffolds with dimensions of ~10 × 10 × 2 mm were produced. Prior to cell-seeding, the porous scaffolds were sterilized with 70% ethanol under ultraviolet (UV) irradiation overnight, followed by air-drying to remove ethanol in a clean bench. To roughly evaluate the effect of CaP content on biocompatibility of the PCL–CaP composite scaffolds, the static cell seeding method was employed [49]. The cell suspension was pipetted on the top of each scaffold, and thus a portion of the cells would preferentially adhere to the surfaces of the filaments rather than their interior.

The preincubated cells were plated at a density of 3 × 10^4^ cells/mL and 1 × 10^4^ cells/mL for the initial cell attachment and proliferation tests, respectively. The MC3T3-E1 cells were cultured in a humidified incubator in an atmosphere containing 5% CO_2_ at 37 °C. A minimum essential medium (α-MEM: Welgene Co., Ltd., Seoul, Korea) supplemented with 10% fetal bovine serum (FBS), 1% penicillin-streptomycin, 10 mM β-glycerophosphate (Sigma), and 10µg mL^−1^ ascorbic acid was used as the culturing medium. 

After 1, 3, and 5 days of cell culturing, the morphologies of the cells on the macro/micro-porous PCL and PCL–CaP composite scaffolds were examined by confocal laser scanning microscopy (CLSM; C1 PLUS, Nikon, Tokyo, Japan). For these CLSM observations, the cultured cells were fixed 4% paraformaldehyde, washed in PBS (phosphate buffered saline), and permeabilized with 0.1% Trion X-100 in PBS for 5 min. Subsequently, actin and cell nuclei were stained with fluorescent phalloidin (Alexa Fluor 555 phalloidin, Invitrogen, USA) and 4′,6-diamidino-2-phenylindole (DAPI; ProLong Gold antifade reagent with DAPI, Invitrogen, USA), respectively. The stained substrates were placed on a cover slide, and the cell morphologies were observed.

After 5 days of cell culturing, the cell proliferation rate was examined using a MTS (methoxyphenyl tetrazolium salt) assay with 3-(4, 5-dimethylthiazol-2-yl)-5-(3-carboxymethoxyphenyl)-2-(4-sulfophenyl)-2H-tetrazolium (MTS, Promega, Madison, WI, USA) for mitochondrial reduction. The quantity of the formazan product, which would be directly proportional to the number of living cells in the culture, was measured by the absorbance at 490 nm using a microplate reader. After 3 h of cell culturing, approximately 25% of the initial cells was observed to adhere to the surfaces of the filaments.

### 2.5. In Vitro Apatite-Forming Ability Evaluation

The in vitro apatite-forming ability of the hierarchical macro/micro-porous PCL–CaP composite scaffolds produced with various CaP contents (10 and 20 wt%) was characterized using simulated body fluid (SBF) solution [50]. For comparison purposes, the PCL scaffold was also tested. The porous scaffolds were immersed in the SBF solutions and then placed inside an incubator at 37 °C for 7 days. The formation of apatite layers on the porous scaffolds was examined by FE-SEM and energy dispersive spectrometry (EDS). 

### 2.6. Mechanical Properties Tests

The mechanical properties of the hierarchal macro/micro-porous PCL–CaP composite scaffolds produced with various CaP contents (0, 10, and 20 wt%) were characterized by tensile and compressive strength tests. The porous scaffolds with dimensions of ~10 × 30 × 2 mm were elongated at a cross-head speed of 3 mm/min using a screw-driven load frame (Oriental Testing Machine Co., Korea). For compressive strength tests, the porous scaffolds with dimensions of ~10 × 10 × 2 mm were compressed at a cross-head speed of 1 mm/min. During the tests, the stress versus strain responses of the scaffolds were recorded. The tensile strength, tensile modulus, and compressive modulus were calculated from the stress-strain curves. All measurements were carried out five times for each scaffold to obtain the mean and standard deviation.

### 2.7. Statistical Analysis

Experimental data were expressed as mean ± standard deviation. Statistical analysis was performed using a one-way analysis of variance (ANOVA) with a Tukey’s post-hoc comparison. A *p* value < 0.05 (*) was considered significant.

## 3. Results and Discussion

### 3.1. Utility of Camphene as Pore-Regulating Agent

**Figure 2:**
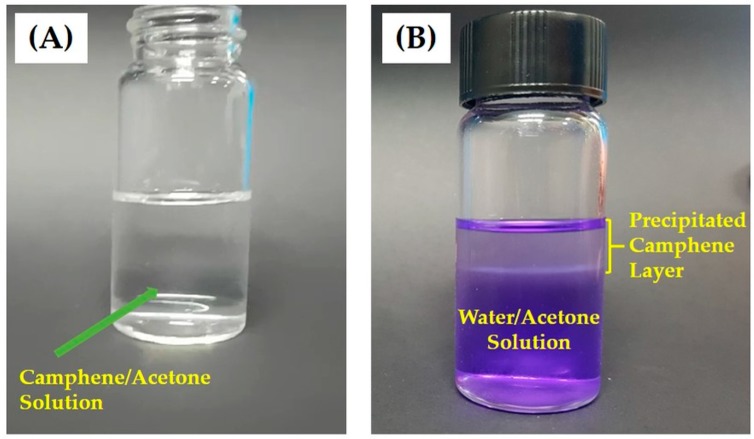
Optical images of (**A**) the camphene–acetone solution and (**B**) the precipitated camphene layer after immersion of the camphene–acetone solution in water. The arrow in Figure 2B indicates the precipitated camphene layer.

We employed solid camphene as the pore-regulating agent to produce microporous PCL filaments with highly porous surfaces, since camphene was highly soluble in the solvent (acetone) yet insoluble in the coagulation medium (water). Figure 2A,B show the representative optical images of the camphene–acetone solution and precipitated camphene layer after immersion of the camphene–acetone solution in water, respectively. Unlike conventional particulate porogens (e.g., salts), the camphene could be completely dissolved in acetone, resulting in a clear solution (Figure 2A). In addition, when this camphene–acetone solution was immersed in water, the camphene phase, indicated by the arrow, could be precipitated though the exchange of acetone and water (Figure 2B). Note that the precipitated camphene can be removed by freeze-drying, thereby creating micropores. 

### 3.2. Effect of Camphene Content on Micropore Generation

**Figure 3:**
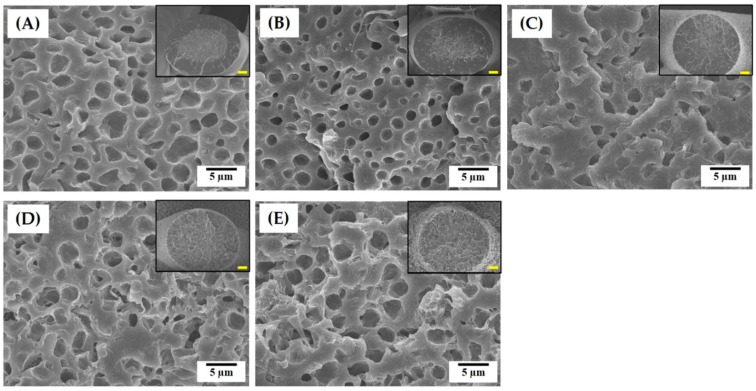
Representative field emission -SEM images of the microporous structures of the PCL filaments produced using various camphene contents: (**A**) 0 wt%, (**B**) 20 wt%, (**C**) 30 wt%, (**D**) 40 wt%, and (**E**) 50 wt%. Insets in Figure 3A–E show the cross-sectional morphologies of the PCL filaments.

Regardless of camphene content, all of the PCL solutions could be effectively used as the feedstock for phase separation-assisted 3D plotting, and thus solid PCL filaments with a circular geometry could be obtained (inset in Figure 3A–E). However, their microporous structures were strongly affected by the camphene content. Figure 3A–E show representative FE-SEM images of the PCL filaments produced using various camphene contents (0, 20, 30, 40, and 50 wt%). Without the addition of camphene, the PCL filament presented a thick dense shell (inset in Figure 3A) with a microporous core (Figure 3A). A similar core/shell structure was observed for the PCL filament produced with the relatively low camphene content of 20 wt% (Figure 3B). On the other hand, when higher camphene content (30, 40, and 50 wt%) was used, all PCL filaments presented microporous structures with negligible formation of a dense layer (Figure 3C–E). 

The present approach utilizes a nonsolvent-induced phase separation (NIPS) process to create the microporous structure. Therefore, the generation of the micropores should be strongly influenced by the interaction of the solvent (acetone) and camphene with the nonsolvent (water). Without the addition of camphene, the acetone in the PCL solution is extracted very rapidly in water, forming a dense layer on the surface of the PCL filament and leaving an inner core with a microporous structure. However, the camphene dissolved in the PCL solution also precipitates in water, reducing the exchange rate of acetone for water, and thus retarding the formation of a dense outer layer. 

### 3.3. Surface Microporous Structures of PCL Filaments

**Figure 4:**
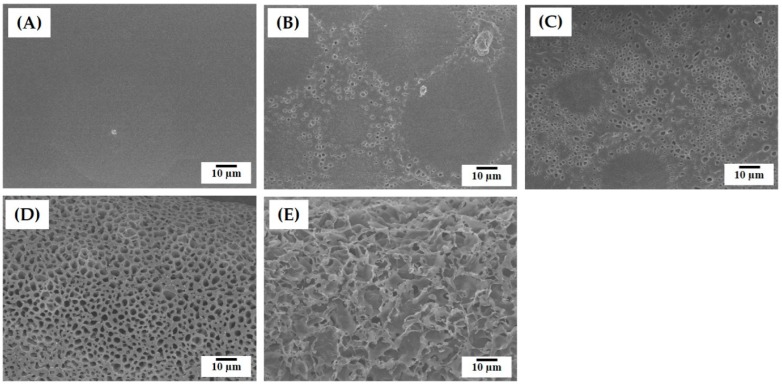
Representative FE-SEM images of the surface morphologies of the PCL filaments produced using various camphene contents: (**A**) 0 wt%, (**B**) 20 wt%, (**C**) 30 wt%, (**D**) 40 wt%, and (**E**) 50 wt%.

One of the most striking advantages of the present approach—the use of camphene as a pore-regulating agent—is the ability to create highly microporous surfaces. Figure 4A–E show representative FE-SEM images of the surface of the PCL filaments produced with various camphene contents (0, 20, 30, 40, and 50 wt%). Without the addition of camphene, the PCL filament presented a dense surface structure (Figure 4A). However, when a camphene content of 20 wt% was used, the surface showed a mixture of the relatively dense and porous regions (Figure 4B). A similar microstructure was observed for the PCL filament produced with 30 wt% camphene, but the fraction of the porous region was markedly increased (Figure 4C). On the other hand, the PCL filaments produced with higher camphene contents (40 and 50 wt%) showed very different microstructures (Figure 4D,E). That is, numerous micropores were uniformly generated on the surfaces. However, the PCL filament produced with a highest camphene content of 50 wt% displayed a highly porous structure (Figure 4E). Significantly, such microporous surfaces would be beneficial to facilitate scaffold-cell interactions such as cell attachment, proliferation, and differentiation [25,26,27]. 

### 3.4. Internal and Surface Microposities of PCL Filaments

The fractions of microporosities of the PCL filaments produced using various camphene contents (0, 20, 30, 40, and 50 wt%) were roughly computed by ImageJ software based on their FE-SEM images. The internal microporosity increased with an increase in camphene content, as summarized in Table 2. The PCL filaments obtained using low camphene contents had dense shells, thus resulting in low porosities, although they had a number of micropores within their core region. In addition, the pure PCL filament showed negligible porosity due to is dense shell, while the PCL filaments obtained using high camphene contents (40 and 50 wt%) showed very high surface microporosities. 

### 3.5. Chemical Structure of PCL Filaments

**Figure 5:**
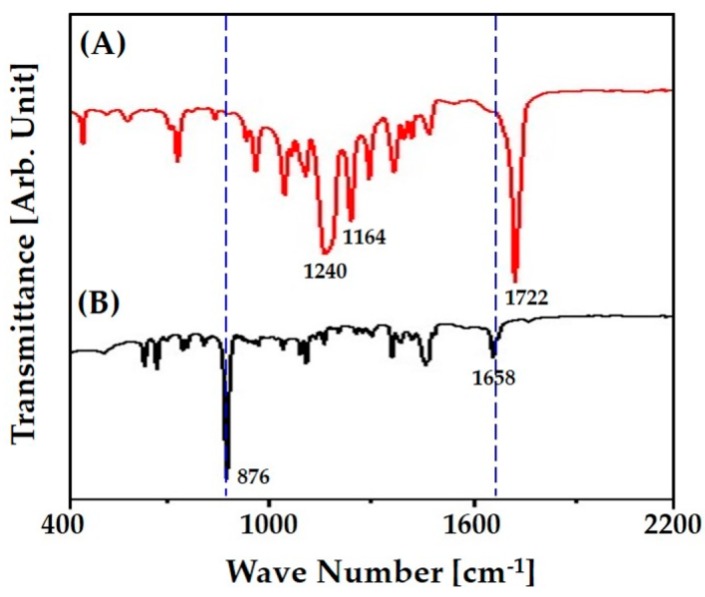
FT-IR spectra of (**A**) the PCL scaffold and (**B**) camphene.

The possibility of camphene residue in the produced PCL scaffold even after freeze-drying was carefully characterized by FT-IT analyses, as shown in Figure 5A,B. The PCL scaffold revealed the typical characteristic peaks associated with PCL: a strong band at 1722 cm^−1^ assigned to carbonyl stretching, and those at 1240 cm^−1^ and 1164 cm^−1^ attributed to the symmetric and asymmetric stretching of the C-O-C group, respectively (Figure 5A) [51]. However, no peaks associated with camphene—associated with the vibration of C=C group at 1658 cm^−1^ and associated with aromatic C-H out of plane at 876 cm^−1^ (Figure 5B) [52]—were observed for the PCL scaffold. This finding suggests that the camphene in the as-plotted PCL scaffold could be completely removed by freeze-drying, while the chemical structure of PCL polymer could be preserved. In addition, it was observed that camphene has negligible cytotoxicity when tested using in vitro cell culture models [53]. Thus, it reasonable to suppose that the porous PCL and PCL–CaP composite scaffolds produced using our approach had good cytocompatibility and biocompatibility in vitro and in vivo.

### 3.6. Hierarchical Porous Structures of PCL–CaP Composite Scaffolds

**Figure 6:**
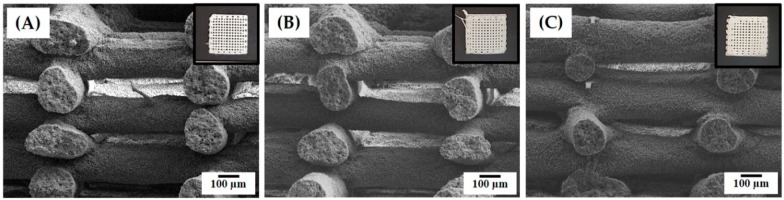
Representative FE-SEM images of the hierarchical macro/micro-porous porous PCL–CaP composite scaffolds produced using different CaP contents: (**A**) 0, (**B**) 10, and (**C**) 20 wt%. Insets in Figure 5A–C show optical images of the produced PCL–CaP composite scaffolds.

To enhance the biological and mechanical functions of the PCL scaffolds for bone scaffold applications, we employed the bioactive and stiff CaP ceramic as reinforcement [5,52]. It should be noted that the PCL–camphene–acetone solution with a camphene content of 50 wt% was used to prepare the PCL–CaP solutions in order to create hierarchical macro/micro-porous PCL–CaP composite scaffolds composed of filaments with internal and surface micropores. The CaP particles could be uniformly dispersed in PCL solution by magnetic stirring. This allowed the production of hierarchical macro/micro-porous PCL–CaP composite scaffolds with various CaP contents (0, 10, and 20 wt%), as shown in the insets in Figure 6A–C. All of the produced PCL–CaP scaffolds exhibited PCL–CaP filaments deposited in a controlled fashion and strongly bonded together, resulting in controlled macroporous structures (Figure 6A–C). 

**Figure 7:**
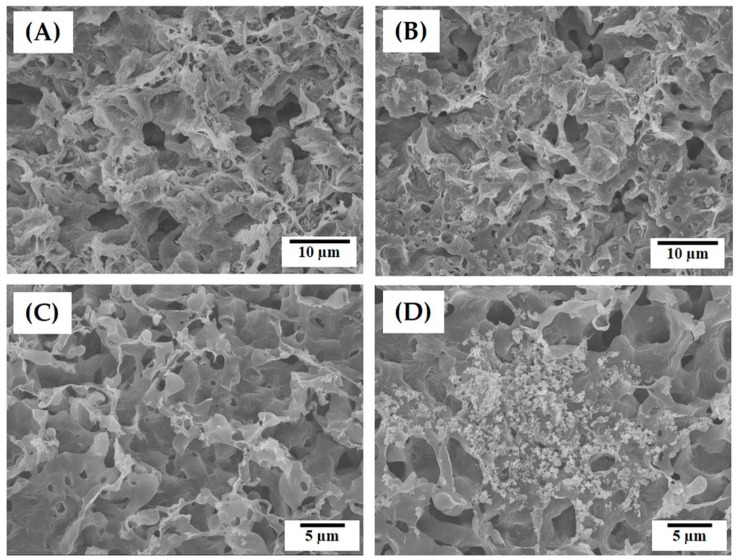
Representative FE-SEM images of the hierarchical macro/micro-porous porous PCL–CaP composite scaffolds produced using different CaP contents of (**A,C**) 10 wt%, and (**B,D**) 20 wt%, showing their (**A**,**B**) internal and (**C**,**D**) surface structures.

The internal and surface microporous structures of the PCL–CaP composite filaments produced using the CaP contents of 10 and 20 wt% were more closely examined by FE-SEM, as shown in Figure 7A–D. Both PCL–CaP composite filaments showed microporous structures, while PCL–CaP composite walls were uniformly created (Figure 7A,B). However, the morphologies of the micropores were slightly different from those produced without the addition of CaP (see Figure 3E). These changes would be attributed to the increased viscosity of PCL–CaP solutions, affecting the phase separation behavior during the 3D plotting process in the coagulation medium. However, it was observed that the outer parts of the PCL–CaP composite filaments were less porous than their inner parts. On the other hand, both PCL–CaP composite filament showed highly microporous surfaces (Figure 7C,D). In addition, the CaP particles were well distributed throughout the PCL–CaP composite walls. This finding suggests that the addition of the CaP particles does not hinder the formation of highly microporous surfaces, and thus the present approach can be used to produce hierarchical macro/micro-porous PCL–CaP composite scaffolds featuring highly microporous surfaces.

The internal microporosities of the PCL–CaP composite filaments with CaP contents of 10 and 20 wt% were ~18.4 and 20.9 vol%, respectively, which were slightly lower than that of the pure PCL filament (see Table 2). In addition, surface microporosities of ~30.6 and 29.6 vol% were observed for the PCL–CaP composite filaments, with CaP contents of 10 and 20 wt%, respectively. These findings suggests that the addition of the CaP particles in the PCL solution would slightly affect the phase separation of PCL, but still allow the formation of highly microporous surfaces.

**Figure 8:**
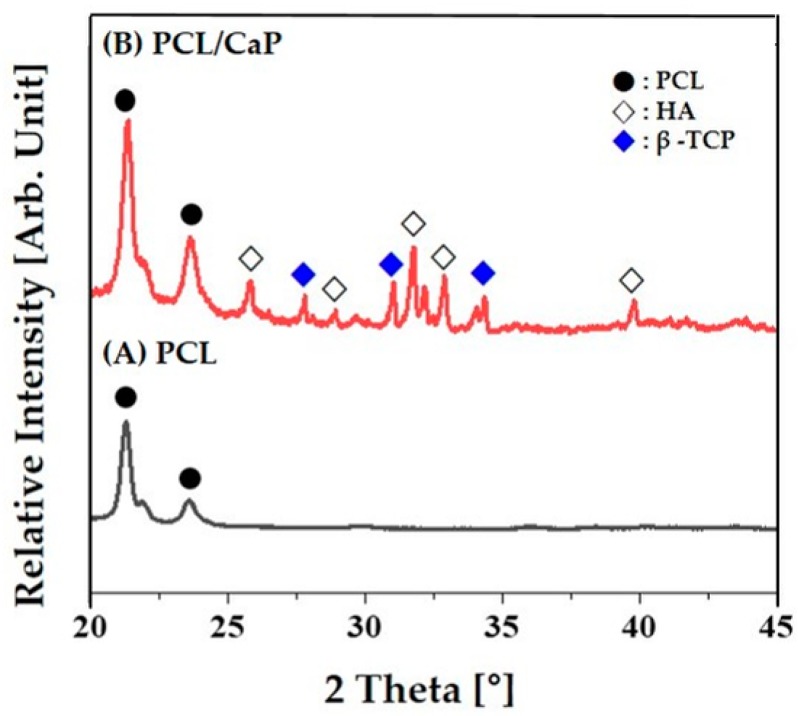
Representative XRD pattern of (**A**) the PCL and (**B**) PCL–CaP composite scaffold produced using a CaP content of 20 wt%.

The presence of the CaP particles in the PCL–CaP composite scaffolds was confirmed by XRD. Figure 8A,B show representative XRD patterns of the PCL and PCL–CaP composite scaffold produced using a CaP content of 20 wt%. The PCL scaffold revealed peaks corresponding to the PCL polymer owing to its semi-crystalline structure (Figure 8A) [1,2]. In this study, we employed biphasic calcium phosphate (BCP) comprised of HA and TCP as reinforcement, since it can provide superior biological and mechanical functions to the monophasic ceramics of either HA or TCP [54]. Thus, peaks associated with both the crystalline HA and TCP phases were observed for the PCL–CaP composite scaffold. This finding suggests that the use of acetone and camphene does not affect the crystalline phases of the BCP, and thus the excellent biocompatibility and bioactivity of BCP can be preserved.

### 3.7. In Vitro Biocompatibility and Apatite-Forming Ability of PCL–CaP Composite Scaffolds

**Figure 9:**
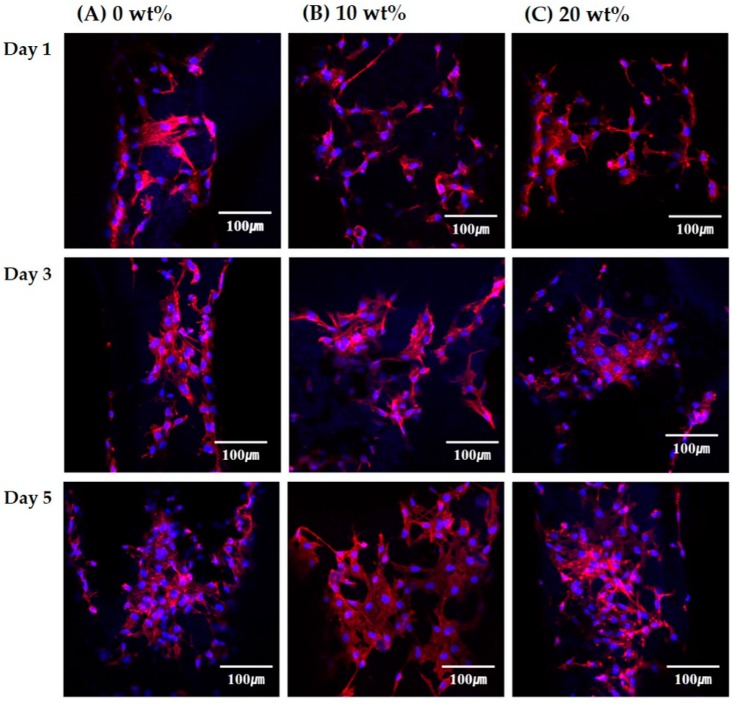
Representative confocal laser scanning microscopy (CLSM) images of the MC3T3-E1 cells on the hierarchical macro/micro-porous PCL–CaP composite scaffolds produced using various CaP contents of (**A**) 0 wt%, (**B**) 10 wt%, and (**C**) 20 wt% at 1, 3, and 5 days.

The effect of CaP content on the in vitro biocompatibility of the hierarchical macro/micro-porous PCL–CaP composite scaffolds was examined. Figure 9A–C show representative CLSM images of the MC3T3 cells attached to the hierarchical porous PCL–CaP composite scaffolds produced using different CaP contents (0, 10, and 20 wt%) after various durations of cell culturing. The red and blue colors represent the actin and nucleus, respectively. After 1 day of cell culturing, all of the PCL and PCL–CaP composite scaffolds presented that the cells adhered to and spread actively across their surfaces. However, compared to the PCL scaffold (Figure 9A), the PCL–CaP composite scaffolds presented more vigorously organized actin stress fibers (Figure 9B,C), which are one of the main components of the cytoskeleton and play a critical role in the control of many aspects of cellular activities. In addition, the cells continued to grow and the number of the cells increased with an increase in time of cell culturing (3 days and 5 days). This finding suggests that all the PCL–CaP composite scaffolds had excellent osteoblast activity.

**Figure 10:**
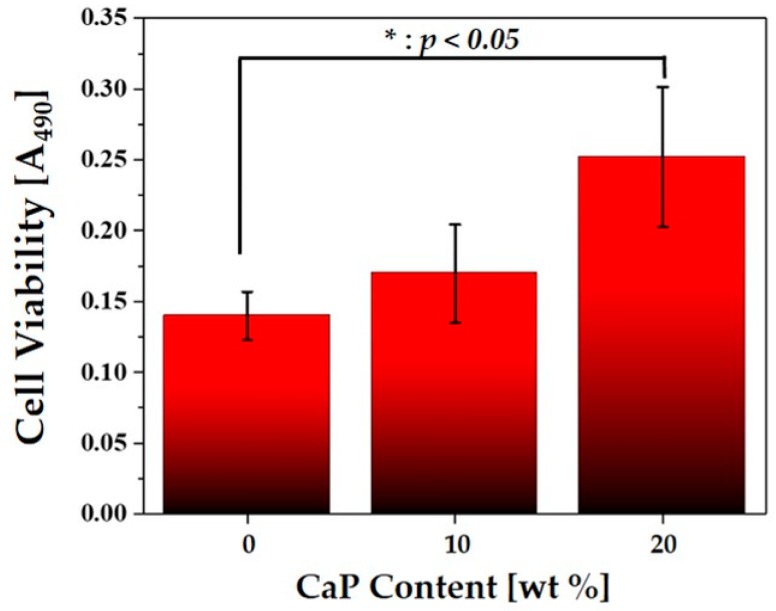
Cell viability of the MC3T3-E1 cells cultured for 5 days on the macro/micro-porous PCL–CaP composite scaffolds produced with various CaP contents (0, 10, and 20 wt%).

The effect of CaP addition on the cell proliferation behaviors was more closely examined by MTS assay, as shown in Figure 10. Cell viability increased with increased CaP content. In addition, the PCL–CaP composite scaffold with a CaP content of 20 wt% showed significantly higher cell viability than the PCL scaffold. The cell densities, computed from the absorbance at 490 nm, were ~1.75 × 10^4^ cells/mL, 2.1 × 10^4^ cells/mL, and 3.2 × 10^4^ cells/mL for the PCL–CaP composite scaffolds produced with CaP contents of 0, 10, and 20 wt%, respectively. This finding suggests that the incorporation of bioactive CaP particles into hierarchical macro/microporous PCL-based scaffolds can significantly enhance their in vitro biocompatibility.

**Figure 11:**
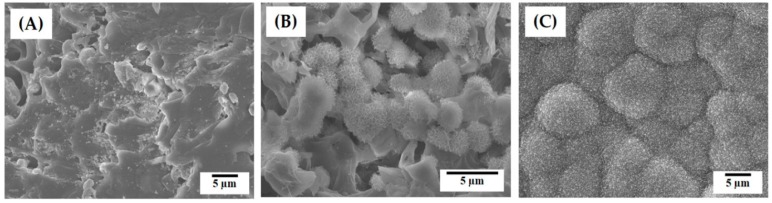
Representative FE-SEM images of the hierarchical macro/micro-porous PCL–CaP composite scaffolds produced with various HA contents after soaking in simulated body fluid (SBF) solution for 7 days: (**A**) 0 wt%, (**B**) 10 wt%, and (**C**) 20 wt%.

The effect of CaP addition on the in vitro apatite-forming ability of the hierarchical macro/micro-porous PCL–CaP composite scaffolds was examined using a simulated body fluid (SBF) test that is an indicator of the in vivo bioactivity of biomaterials [50]. Figure 11A–C present representative FE-SEM images of the hierarchical porous PCL–CaP composite scaffolds produced with various CaP contents (0, 10, and 20 wt%) after soaking in SBF solution for 7 days. The PCL scaffold showed a sign of apatite crystal precipitation, where particulate apatite crystals began to form (Figure 11A). On the other hand, the PCL–CaP composite scaffolds showed vigorous precipitation of apatite nanocrystals on their surfaces (Figure 11B,C). Apatite crystals with tiny flake-like morphology was formed that were similar to those observed for CaP ceramics immersed in the SBF [42,50,54]. In addition, the surfaces of the PCL–CaP composite filaments produced with the highest HA content of 20 wt% were almost fully covered by apatite crystals (Figure 11C). This finding suggests that the inclusion of bioactive CaP particles into PCL polymer can significantly enhance the in vitro bioactivity of the macro/micro-porous PCL–HA composite scaffolds.

**Figure 12:**
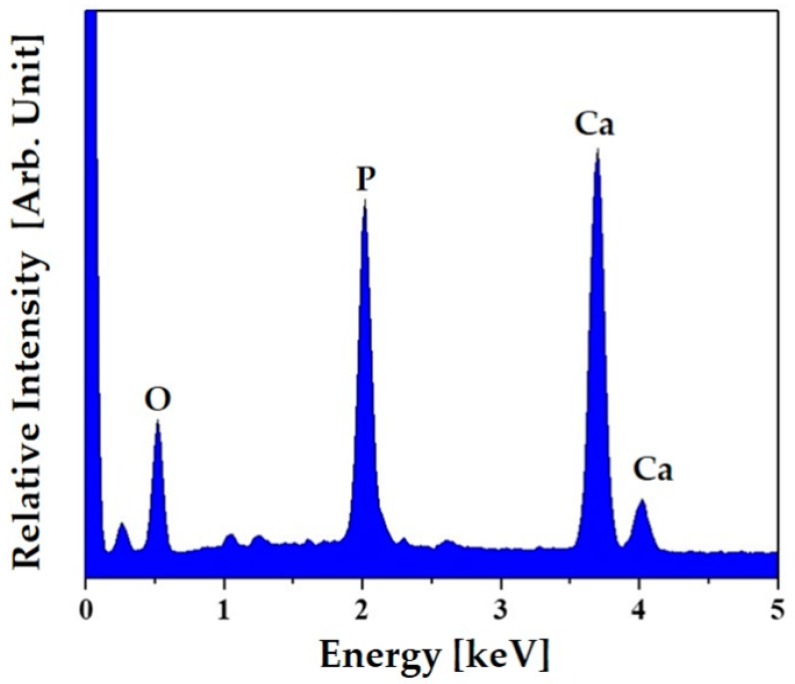
Energy dispersive spectrometry (EDS) spectrum of the apatite crystals formed onto the PCL–CaP composite scaffolds produced with a CaP content of 20 wt%.

The chemical compositions of these apatite crystals were characterized by EDS, as shown in Figure 12. Strong peaks corresponding to Ca, P, and O elements were observed, indicating that the precipitated phase is apatite phase.

### 3.8. Mechanical Properties of PCL–CaP Composite Scaffolds

**Figure 13:**
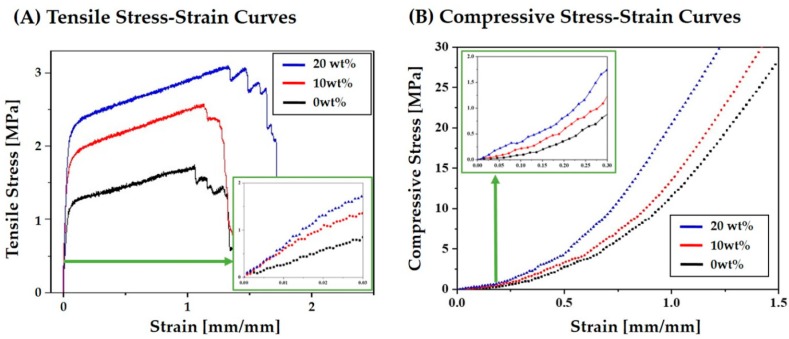
Representative stress versus strain responses of the hierarchical porous PCL–CaP composite scaffolds produced using various CaP contents (0, 10, and 20 wt%) during (**A**) tensile and (**B**) compressive strength tests.

To evaluate the structural integrity of the hierarchical macro/micro-porous PCL–CaP composite scaffolds, their mechanical properties were measured using tensile and compressive strength tests. The representative stress versus strain responses of the hierarchical porous PCL–CaP composite scaffolds produced using various CaP contents (0, 10, and 20 wt%) during the tensile and compressive strength tests are plotted in Figure 13A,B, respectively. All of the porous PCL–CaP composite scaffolds exhibited similar fracture behavior—a characteristic of highly porous polymer scaffolds [6,7].

**Figure 14:**
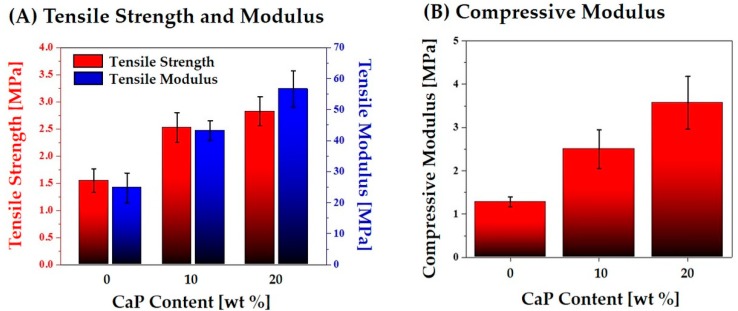
(**A**) Tensile strength and tensile modulus, and (**B**) compressive modulus of the hierarchical porous PCL–CaP composite scaffolds produced using various CaP contents (0, 10, and 20 wt%).

The tensile strength, tensile modulus, and compressive strength were calculated from the stress versus strain responses of the hierarchical porous PCL and PCL–CaP composite scaffolds. As the CaP content increased from 0 to 20 wt%, the tensile strength and modulus increased markedly from 1.55 ± 0.21 MPa to 2.82 ± 0.27 MPa, and from 24.75 ± 4.77 MPa to 56.63 ± 5.86 MPa, respectively (Figure 14A). In addition, compressive modulus increased from 1.28 ± 0.17 MPa to 3.57 ± 0.61 MPa (Figure 14B). This finding suggests that the incorporation of stiff CaP particles can significantly enhance the mechanical properties of the porous PCL scaffolds, while preserving their hierarchical macro/micro-porous structures.

### 3.9. Utility of Present Study 

Our approach—to use camphene as a pore-regulating agent—is a simple and versatile means of producing PCL and PCL-based composite scaffolds with unique hierarchical porous structures simply by utilizing PCL solutions containing camphene dissolved in acetone (solvent) with a phase separation-assisted 3D plotting technique. This innovative approach allows the construction of controlled macroporous structures comprising microporous filaments specifically with open porous structures. In addition, a variety of functional particles, such as calcium phosphate (CaP) ceramics with bioactivity, and Ag and TiO_2_ with antibacterial efficacy, can be incorporated into PCL–camphene–acetone solutions. Thus, PCL and PCL-based composite scaffolds produced using our approach can have unique hierarchical macro/micro-porous structures and find very useful applications in diverse fields [55,56] including bone tissue engineering [57], cardiac tissue engineering [58], and skeletal muscle tissue regeneration [59].

## 4. Conclusions

Hierarchical macro/micro-porous PCL and PCL–CaP composite scaffolds with highly microporous surfaces were produced by a phase separation-based 3D plotting technique using camphene as the pore-regulating agent. Without the addition of camphene, the PCL filament possessed a thick, dense outer layer and a microporous core. In contrast, the use of the camphene contents of 40 and 50 wt% enabled the creation of highly microporous filaments lacking dense outer shells. In addition, PCL–CaP composite scaffolds with the CaP contents of 10 and 20 wt% were successfully produced, which were composed of highly microporous PCL–CaP composite filaments. The incorporation of the CaP particles into the scaffolds significantly enhanced their in vitro biocompatibility and apatite-forming ability. Their mechanical properties, such as tensile strength, tensile modulus, and compressive modulus, were also enhanced markedly compared to the PCL scaffolds. Our approach would be applicable for producing a variety of hierarchical macro/micro-porous polymer-based scaffolds that could be used for various tissue engineering applications.

## Figures and Tables

**Table 1:** Air pressures used to extrude PCL–camphene solutions with different camphene contents (0, 20, 30, 40, and 50 wt%).

Camphene Content [wt %]	0	20	30	40	50
**Air Pressure [kPa]**	10	8	6	4	3

**Table 2:** Internal and surface microporosities of the PCL filaments produced using various camphene contents (0, 20, 30, 40, and 50 wt%).

Porosity	Camphene Content [wt %]				
	0	20	30	40	50
**Internal Porosity [vol%]**	13.2	15.5	17.7	20.4	25.5
**Surface Porosity [vol%]**	0.35	6.2	11.2	30.2	32.5
